# Effect of Painful and Non-Painful Sensorimotor Manipulations on Subjective Body Midline

**DOI:** 10.3389/fnhum.2013.00077

**Published:** 2013-03-14

**Authors:** Jason Bouffard, Martin Gagné, Catherine Mercier

**Affiliations:** ^1^Centre Interdisciplinaire de Recherche en Réadaptation et en Intégration SocialeQuébec, QC, Canada; ^2^Département de Réadaptation, Faculté de Médecine, Université LavalQuébec, QC, Canada

**Keywords:** body perception, spatial attention, neuropathic pain, neglect, egocentric frame of reference

## Abstract

Patients with chronic pain often show disturbances in their body perception. Understanding the exact role played by pain is however complex, as confounding factors can contribute to the observed deficits in these clinical populations. To address this question, acute experimental pain was used to test the effect of lateralized pain on body perception in healthy subjects. Subjects were asked to indicate the position of their body midline (subjective body midline, SBM) by stopping a moving luminescent dot projected on a screen placed in front of them, in a completely dark environment. The effect of other non-painful sensorimotor manipulations was also tested to assess the potential unspecific attentional effects of stimulating one side of the body. SBM judgment was made in 17 volunteers under control and three experimental conditions: (1) painful (heat) stimulation; (2) non-painful vibrotactile stimulation; and (3) muscle contraction. The effects of the stimulated side and the type of trial (control vs. experimental condition), were tested separately for each condition with a 2 × 2 repeated measures ANOVA. The analyses revealed a significant interaction in both pain (*p* = 0.05) and vibration conditions (*p* = 0.04). *Post hoc* tests showed opposite effects of pain and vibration. Pain applied on the right arm deviated the SBM toward the right (stimulated) side (*p* = 0.03) while vibration applied on the left arm deviated the SBM toward the right (not stimulated) side (*p* = 0.01). These opposite patterns suggest that the shift in SBM is likely to be specifically linked to the stimulation modality. It is concluded that acute experimental pain can induce an SBM shift toward the stimulated side, which might be functionally beneficial to protect the painful area of the body. Interestingly, it appears to be easier to bias SBM toward the right side, regardless of the modality and of the stimulated side.

## Introduction

Patients with chronic pain often show disturbances in their body perception. For example, alterations in the perceived size or shape of the painful body parts have been reported in complex regional pain syndrome (CRPS) patients (Moseley, 2005; Lewis et al., 2007, 2010; Peltz et al., 2011) and low back pain patients (Moseley, 2008). In a hand size estimation task, CRPS patients judge their affected hand to be larger than its actual size (Moseley, 2005; Peltz et al., 2011). When asked to place their spine on a drawing of a back, patients with low back pain tend to draw it toward the painful side (Moseley, 2008). Deafferentation, as in the case of amputation, is also associated with altered body perception. Most amputees continue to feel the presence of their amputated limb (phantom limb) but its size, posture, and integrity are often altered (Giummarra et al., 2010). In addition to these persistent sensations of their lost limb, most amputees also experience pain in their missing limb (Ephraim et al., 2005).

As amputation is an extreme case of sensorimotor alteration, more subtle abnormalities in sensorimotor processing are observed in other chronic pain populations. Alteration of complex tactile functions such as tactile acuity (Moseley, 2008; Wand et al., 2010; Peltz et al., 2011), graphesthesia (Wand et al., 2010), or tactile stimuli localization (Forderreuther et al., 2004) have been demonstrated. Similarly, proprioception can be impaired as illustrated by poorer performance in a limb positioning task in various chronic pain populations (Pinsault et al., 2008; Lewis et al., 2010; Anderson and Wee, 2011). Furthermore, chronic pain patients tend to move more slowly (Schilder et al., 2012). Even their ability to imagine movements, a process known to at least partially solicit the same brain areas as those involved in movement production, is impaired, as illustrated by their performance in the laterality judgment task (Schwoebel et al., 2001, 2002; Moseley, 2004, 2008; Coslett et al., 2010a,b; Mercier, 2012). Together, these observations suggest that pain is related, to some extent, to perturbations in sensorimotor processing and may lead to a distortion of body representations. Neurophysiological data support this view, as pain intensity has been shown to be linked to the extent of reorganizations in the primary sensorimotor cortex in different population of patients with chronic pain (Flor et al., 1995; Karl et al., 2001; Lotze et al., 2001; Schwenkreis et al., 2009; Wrigley et al., 2009; Henry et al., 2011).

In a recent study, Moseley et al. (2009) showed that, in order to perceive tactile stimulations on both arms as simultaneous, CRPS patients have to receive the stimulation on the affected arm a few milliseconds before the stimulation on the unaffected arm. This observation was interpreted as neglect of the affected arm. However, when the same procedure was tested while patients had their arms crossed the stimulation had to be applied a few milliseconds earlier on the unaffected arm to be perceived as simultaneous. Thus, it suggests that the neglect is not related to the affected arm *per* *se* but to the hemispace where the painful limb normally lays. In other words, it suggests that the association between altered sensorimotor processing and pain is not related (or at least not exclusively related) to an altered somatotopical body representation, as plastic changes observed in the primary sensorimotor cortices might suggest. Rather, it seems to imply broader, multimodal information processing related to the egocentric framing of space (Legrain et al., 2012).

A series of studies by Sumitani et al. (2007a,b) and Uematsu et al. (2009) supports this view. They asked CRPS patients to align the position of a luminescent dot on their perceived body midline (subjective body midline, SBM) in a dark environment. They showed that patients systematically judged their SBM toward their painful side. This illustrates that the alignment of the proprioceptive and visual maps, which is important in maintaining the integrity of the egocentric frame of reference, is altered in these patients.

However, research with clinical pain populations may involve confounding factors, not associated directly with pain (for example disuse), that have the potential to influence sensorimotor processing. Although, as observed in pathologic pain, experimental pain has been shown to influence body perception (Gandevia and Phegan, 1999), other behavioral observations showed some discrepancies between clinical and experimental pain (Moseley et al., 2005). The objective of the present study was to evaluate the effect of acute experimental pain on the perception of body midline, which strongly relies on the proper integration of somatosensory and visual inputs. According to Sumitani’s studies with CRPS patients (Sumitani et al., 2007a,b; Uematsu et al., 2009), we hypothesized that SBM should deviate toward the stimulated side in the presence of experimental pain. The effect of other non-painful sensorimotor manipulation was also tested to assess for potential unspecific attentional effects of stimulating one side of the body.

## Materials and Methods

### Subjects

Seventeen subjects participated in the study (nine females; mean age 26.9, SD 5.6). All were right handed according to the Edinburgh handedness questionnaire (Oldfield, 1971) and all had normal or corrected to normal vision. All subjects gave their written informed consent prior to participating in the experiment, which was approved by the Ethics Committee of the Institut de Réadaptation en Déficience Physique de Québec and conformed to the ethical aspects of the *Declaration* *of* *Helsinki*.

### Experimental procedure

**Figure 1:**
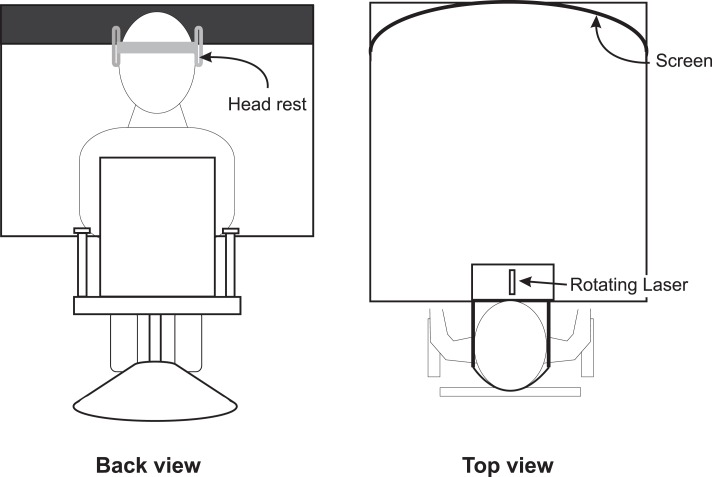
**Experimental set-up used to test the subjective body midline**. Note that the screen on which the laser was projected was curved to ensure that the speed of the projected laser dot remained constant.

The SBM was tested in a dark environment to ensure that subjects had no visual spatial reference. A schema of the set-up is shown in Figure 1. Subjects sat in front of the experimental device and stared at a black screen. The subject was seated in a hydraulic chair (allowing vertical and lateral positioning). Once in place, the subject’s head was restrained from movement in a headrest, fixed on the center of the experimental device (see Figure 1). A pointing laser mounted on a rotating motor projected a red dot onto the black screen. The width of the screen was 1.15 m, which corresponds to 60° of eccentricity (both lateral edges at 30° of eccentricity). The center of the screen was given the position 0° flanked with positive values on the right and negative values on the left. To judge the SBM, the pointer lit up and a red dot appeared on the screen. For each trial, the initial dot position was randomly determined. It was located at eye level, either on the right or on the left of the screen’s center, between 15° and 25° of eccentricity. In the same conditions, half of the trials started from the right and the other half started from the left, with symmetrical positions. After its apparition, the red dot started moving toward the center at a constant speed of 3°/s. Subjects indicated their perceived SBM by stopping the red dot with a verbal command, consisting in briefly blowing in a microphone positioned immediately before the subject’s mouth. The microphone signal was interfaced with the laser controlling system. To determine the SBM, the onset of the voice signal was identified and the position of the red dot at that moment was assessed by monitoring the rotation of the shaft on which the pointing laser was mounted. The signal from an optical encoder, detecting the shaft rotation, and the microphone were fed through an analog-to-digital converter (CED 1401 interface; Cambridge Electronic Design, Cambridge, UK) and then sent to a personal computer. Data were compiled with Spike 2 (Cambridge Electronic Design, Cambridge, UK).

### Conditions

The effect of pain and of two non-painful sensorimotor manipulations, vibrotactile stimulation and muscle contraction, on the SBM was tested on both sides of the body. Each condition was tested in a separate block (for a total of six experimental blocks; three conditions × two sides). At the beginning of each block, the subject’s head was positioned in the headrest and the shoulders were aligned parallel to the screen. The subject was prompted to remain as still as possible throughout the block. Sixteen trials were tested in each block. No stimulation was applied in the first six trials and they served as controls for that particular block. In the remaining 10 trials (test trials), the appropriate stimulation was applied during each trial. Each trial was separated by 30 s. The effect of all three conditions on SBM judgment were first tested on one arm and then tested on the other arm. The order in which each arm was tested was counter-balanced across subjects as well as the order of tested conditions on each arm.

The pain condition consisted in applying a controlled thermal stimulation via a 30 × 30 mm thermode (Pathway Model ATS, Medoc advanced medical system, Israel) placed on the ventral face of the forearm. Before beginning the SBM assessments, we determined the pain threshold for each arm. To do this, the thermode temperature was initially set at 33°C and gradually increased at a rate of 1.5°C/s. The subject was instructed to push a button to stop the thermal stimulation when he/she felt that the stimuli switched from a sensation of warmth to a sensation of pain. This procedure was repeated three times and the mean of the temperature measured at each button press was calculated and considered as the pain threshold. The default stimulation temperature used in the SBM measurement trials was set at 0.5°C above measured threshold. In these trials, the thermode temperature started at 33°C and rose up at a rate of 3.5°C/s until it reached the stimulation temperature, plateaued for 10 s, and fell back to 33°C at a rate of 8°C/s. The pain sensation had to be considered as moderate by the subject across the whole 10 s. In a few subjects, it has been necessary to slightly re-adjust the temperature to obtain a moderate pain level over the 10-s period. For the SBM judgment, the red dot appeared on the screen at the moment the painful stimulation temperature was reached. The duration of the painful stimulus was not influenced by the subject’s response time.

In the vibration condition, a custom made vibrator was fixed on the ventral face of the midportion of forearm with a Velcro strap. The vibration frequency was set at 25 Hz. It is important to note that such parameters do not induce illusions of movement (typically induced while vibrating the tendons at frequencies between 60 and 80 Hz (Roll and Vedel, 1982). During a trial, vibration started 4 s before the red dot appeared on the screen and ended when the subject stopped the laser.

The muscle contraction condition consisted in supporting a weight (conventional dumbbell) with the forearm in a neutral position with the elbow flexed at about 90°. Before beginning the experimental procedure, each biceps maximal voluntary contraction was measured by means of surface electromyographic (EMG) recordings during an isometric contraction. The subject was then required to hold different weights and the weight generating an EMG corresponding to 15% (±5%) of the maximal contraction was retained for the experimental task. During the muscle contraction condition, the weight was lifted for 4 s before the red dot was presented and started moving on the screen. The subject was instructed to hold the weight until the SBM judgment was completed and to rest afterward. The rational for applying vibration and to lift the weight about 4 s before starting the trials was to match the rise time of the temperature in the painful condition.

### Data analysis

In each block of trials, the mean of the 6 control trials and the mean of the 10 test trials were calculated for statistical analysis. In order to avoid a potential bias that could have been introduced by a slight difference in the head placement across blocks, the SBM measured during a painful/non-painful sensorimotor manipulation in one block was always compared to the SBM measured during control trials of the same block. As the aim of the study was to assess the individual effect that each sensorimotor manipulation had on the SBM, rather than to quantitatively compare the effect of each manipulation relative to the others (as it is impossible to match the intensity of a muscle contraction or of a non-painful stimuli to that of a painful stimuli), a distinct analysis was performed for each condition. As such, for each experimental condition, the effect of the stimulated side (right vs. left) and the effect of the type of trial (control vs. test) were tested with a 2 × 2 (side × trial type) repeated measures ANOVA computed with SPSS 13.0 software (without correction for multiple testing, given that a different set of data was employed for each analysis). When a significant interaction effect was found, paired *t*-tests [corrected for multiple testing using a Hochberg procedure (Olejnik et al., 1997)] were used for pre-planned comparisons between control and test trials measured on the same tested body side.

In order to compute group descriptive statistics on the effect of each experimental condition, normalized SBM was calculated for each subject in each experimental block. This was done by subtracting, for each block, the mean SBM measured in control trials from the mean SBM measured in test trials (°test − °control). Thus, a normalized SBM with a positive value indicates that the sensorimotor stimulation shifted the SBM to the right and conversely, a negative value indicates a shift to the left.

## Results

The mean stimulation temperature used during the SBM judgment was 47.2°C (SD 1.9) on the right arm and 47.5 (SD 1.5) on the left which is not statistically different [*t*_(16)_ = −0.97, *p* = 0.35].

**Figure 2:**
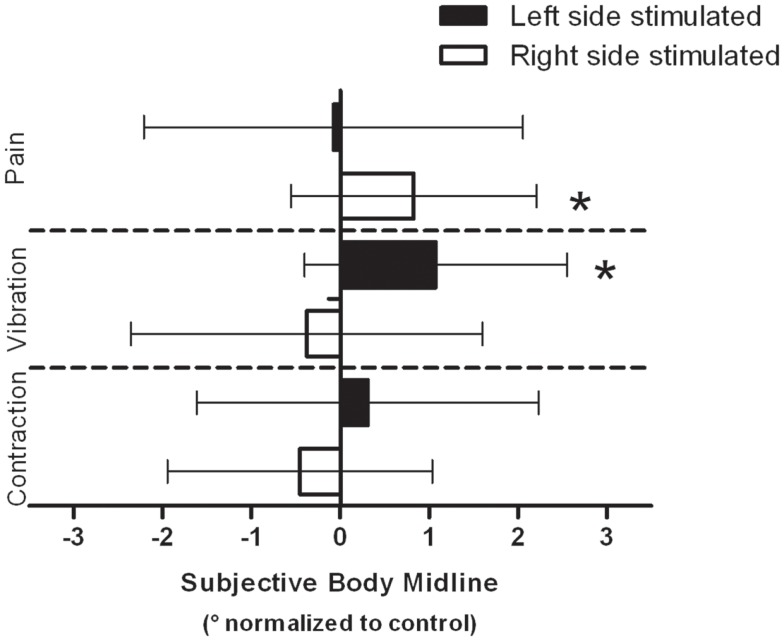
**Group data for each modality are presented**. Data are normalized to their respective controls (see Materials and Methods). Negative values indicate a shift of the SBM position toward the left and conversely, a positive value indicates a shift toward the right. Error bars indicate the standard deviation on the mean normalized data. *Indicates a statistically significant difference between the test SBM (e.g., with pain/vibration/contraction) and the control SBM measured in the same block.

**Figure 3:**
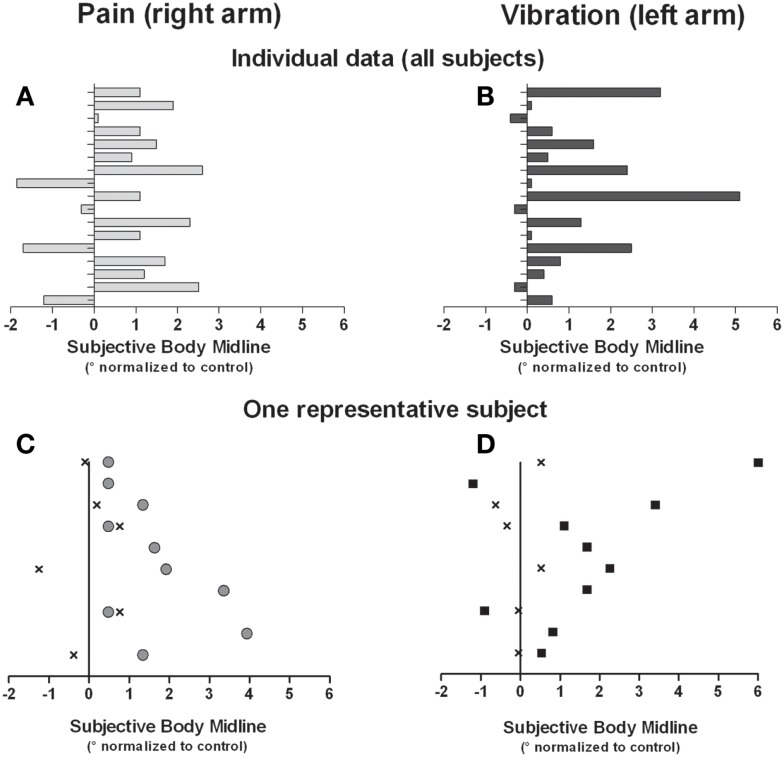
**Individual data for all subjects are shown for the right pain condition (A) and the left vibration condition (B)**. In addition, data from one subject in the right pain condition **(C)** and the left vibration **(D)** are shown. Data in C and D were each obtained in a single block, and each dot represents a single trial. “X” indicates trials in the control condition (i.e., no stimulation, trials 1–6 of a given block), gray circles in right pain condition, and black squares in left vibration condition (respectively trials 7–16 of a given block). All data are normalized against the average SBM obtained in the control condition.

Group means of the normalized SBM positions for each condition are presented in Figure 2. In the pain condition, the ANOVA revealed an interaction effect [*F*_(1,16)_ = 4.466, *p* = 0.05]. *T*-tests showed that when the painful stimulus was applied over the right arm, the SBM was significantly deviated to the right when compared to control trials [*t*_(16)_ = −2.473, *p* = 0.03]. No such effect was found when the stimulus was applied over the left arm [*t*_(16)_ = 0.148, *p* = 0.90]. An interaction effect was also detected with the ANOVA in the vibration condition [*F*_(1,16)_ = 5.046, *p* = 0.04]. *T*-tests revealed that vibrations applied to the left arm deviated the SBM to the right when compared to control trials [*t*_(16)_ = −3.003, *p* = 0.01]. The effect was not found when the stimulus was applied over the right arm [*t*_(16)_ = 0.785, *p* = 0.4]. Finally, in the contraction condition, there was no significant effect (whether main effect or interaction effect) detected by the ANOVA [*F*_(1,16)_ = 1.380, *p* = 0.26]. Individual results obtained in conditions that yield significant effects indicated that 76% of the subjects had their SBM deviated toward the right when stimulated with pain on their right side and 82% were deviated to the right when vibrotactile stimuli were applied to their left side (see Figure 3).

## Discussion

The primary objective of the present study was to evaluate the effect of acute experimental pain on the perception of body midline, which strongly relies on the integration of somatosensory and visual inputs. Vibrotactile stimulation and muscle contraction were used to control for the effect of non-specific sensory inputs. The results show that both painful and vibrotactile stimulation influence the judgment of the SBM. Importantly, these two stimulation modalities induced opposite effects on the SBM judgment. Indeed, both produced a deviation of the SBM to the right, but this effect was only observed if (a) pain was applied to the *right* side of the body and (b) vibrotactile stimulation was applied to the *left* side of the body. This suggests that the observed deviation in the perceived body midline is modality specific and not simply an unspecific perceptual bias caused by sensory input whatever the stimulation modality used. These results are consistent with those of a few studies reporting that acute pain can interfere with tasks involving multimodal sensory processing. For example, altered perception of the size of the thumb (Gandevia and Phegan, 1999) and perturbations in laterality recognition (Moseley et al., 2005) have been shown when acute nociceptive stimulation is applied to the hand.

Results of clinical studies showed that patients with chronic neuropathic pain (CRPS) tend to judge their body midline toward their painful side (Sumitani et al., 2007b), consistent with our observations in experimental acute pain. Interestingly, they showed that using adaptation to prismatic deviation in these CRPS patients [an “unconscious” way to re-align the distorted visuomotor and proprioceptive maps (Rossetti et al., 2005)] can reduce pain intensity (Sumitani et al., 2007a). This further supports the idea that pain and multimodal sensory processing have mutual interactions. However, the observation that subjective SBM is deviated toward the painful side might appear to be in contradiction with other studies suggesting that patients tend to neglect the side of their body affected by pain (Lewis et al., 2007) and exhibit slower sensorimotor processing on that side. Indeed, CRPS patients have longer reaction time to judge the laterality of pictures corresponding to the affected limb when compared to the unaffected limb (Schwoebel et al., 2001; Moseley, 2004; Moseley et al., 2008; Reinersmann et al., 2010). Another study, with the help of a tactile temporal order task, showed that CRPS patients felt cutaneous stimulations applied over both forearms as simultaneous when the affected arm was actually stimulated first. This apparent discrepancy might reside in part in the nature of the tasks studied. In the latter studies, the experimental manipulations might constitute a “threat” to the painful limb as motor imagery has been shown to increase pain in CRPS patients (Moseley et al., 2008). Also, normally inoffensive tactile stimulation might provoke painful sensations (allodynia) in neuropathic pain populations (Gierthmuhlen et al., 2012). It was proposed that the delayed processing of the sensory information on the painful body part might be a way to protect oneself against painful threats (Moseley, 2004; Moseley et al., 2009). In contrast performing a SBM task does not impact on pain which might explain why in this condition the perception appears to be biased toward the painful side.

Both pain and vibrotactile stimulations led to a rightward deviation of the SBM. This rightward deviation of the SBM is similar to what is observed in patients with a neglect of the left hemispace following a brain lesion (Farne et al., 1998). Indeed, chronic neglect was reported to be three times more frequent in right brain damaged than in left brain damaged patients (Ringman et al., 2004). Thus, dominance might be a factor in the effect we observed (i.e., a deviation of the SBM only toward the right side of the body). Studies in healthy subjects tend to support that right handers showed a systematic bias toward the left side in tasks such as line bisection (Jewell and McCourt, 2000), a phenomenon that was termed pseudoneglect. The presence of such pseudoneglect may hinder the possibility of sensorimotor manipulations to further deviate the SBM toward the left (Kline et al., 2009), or at least make it easier to deviate SBM toward the right.

Voluntary contraction did not induce a shift of the SBM. This might be explained by the fact that when a movement is self-produced, its sensory consequences can be accurately predicted by an internal model (Wolpert et al., 1995). It has been proposed that this prediction can be used to attenuate the sensory effects of the movement (Blakemore et al., 1998) and therefore might cancel the potential impact of sensory feedback on the SBM. Alternatively negative results might also be attributable to limitations related to the methodology. It needs to be kept in mind that all the sensory manipulations were tonic (e.g., stimulation/contraction was maintained for several seconds) because of the time needed to perform the SBM judgment. As such this method does not allow the measurement of the time-course of the effect of a given condition. It can therefore not be excluded that the effect of contraction is too short-lived to be observed using that method. Similarly the effects of pain/vibration on the SBM might vary depending upon the duration of the stimulation. Finally the variability of the SBM measure in the baseline condition might also have led to some negative results, especially given that shifts induced in SBM in healthy subjects are expected to be small. Some of this variability is probably associated to the fact that the subjects had to produce a verbal command in order to stop the moving dot when it was crossing their perceived SBM. Now that we have an estimate of the range of shift of the SBM that can be induced using sensorimotor manipulations, other psychophysical methods, such as two-alternative forced choice for example, might provide a way to improve both the spatial and temporal resolution of the SBM measurement.

## Conclusion

In line with the results of studies in patients with chronic pain, the results of this study indicate that body representation can be influenced by acute painful sensations. They show that acute experimental pain can rapidly shift the SBM bias toward the stimulated side, a phenomenon that might potentially be functionally beneficial to protect the painful body region. This effect was found to be specific to painful stimuli, as the effect of vibration was in the opposite direction. Interestingly, it appears to be easier to bias SBM toward the right side, regardless of the modality and of the stimulated side.

## Conflict of Interest Statement

The authors declare that the research was conducted in the absence of any commercial or financial relationships that could be construed as a potential conflict of interest.
